# Evaluation of COVID-19 Restrictions on Distance Runners' Training Habits Using Wearable Trackers

**DOI:** 10.3389/fspor.2021.812214

**Published:** 2022-01-12

**Authors:** Zoe Y. S. Chan, Rhys Peeters, Gladys Cheing, Reed Ferber, Roy T. H. Cheung

**Affiliations:** ^1^Faculty of Kinesiology, University of Calgary, Calgary, AB, Canada; ^2^Department of Rehabilitation Sciences, Hong Kong Polytechnic University, Kowloon, Hong Kong SAR, China; ^3^School of Health Sciences, Western Sydney University, Campbelltown, NSW, Australia

**Keywords:** wearables, activity monitoring, coronavirus, training intensity, training frequency

## Abstract

The COVID-19 pandemic caused widespread disruption to many individuals' lifestyles. Social distancing restrictions implemented during this global pandemic may bring potential impact on physical activity habits of the general population. However, running is one of the most popular forms of physical activity worldwide and one in which it could be maintained even during most COVID-19 restrictions. We aimed to determine the impact of COVID-19 restrictions on runners' training habits through analyzing the training records obtained from their GPS enabled wearable trackers. Retrospective and prospective data were collected from an online database (https://wetrac.ucalgary.ca). Runners' training habits, including frequency, intensity and duration of training, weekly mileage and running locations were analyzed and compared 9 months before and after the start of COVID-19 restrictions in March 2020. We found that runners ran 3 km per week more (*p* = 0.05, Cohen's *d* = 0.12) after the start of COVID-19 restrictions, and added 0.3 training sessions per week (*p* = 0.03, Cohen's *d* = 0.14). Moreover, runners ran an additional 0.4 sessions outdoors (*p* < 0.01, Cohen's *d* = 0.21) but there was no significant change in the intensity or duration of training sessions. Our findings suggested that runners adopted slightly different training regimen as a result of COVID-19 restrictions. Our results described the collective changes, irrespective of differences in response measures adopted by various countries or cities during the COVID-19 pandemic.

## Introduction

The Severe Acute Respiratory Syndrome Coronavirus 2 (SARS-COV2) and Corona Virus Disease 2019 (COVID-19) caused widespread health impacts and disruption to lifestyles around the globe during 2020. On March 11, 2020, the World Health Organization declared COVID-19 a global pandemic (World Health Organisation, 2020), prompting immediate action from governments to mitigate the spread of the disease in an effort to save lives. Various countries adopted different approaches to slowing the spread of COVID-19, however, almost all countries implemented some form of temporary lockdown measures, with ongoing social distancing restrictions. The impact of such restrictions caused the cancellation of large events and gatherings of people. As countries begin the process of returning to normal life post-COVID-19, it is important to understand the impact that COVID-19 restrictions had on individuals' physical activity and exercise habits.

Running is consistently shown to be one of the most popular forms of exercise, between 7.9 and 13.3% of adults participating in running worldwide (Hulteen et al., 2017). Running as a form of exercise is shown to have a wide variety of health benefits, ranging from improved cardiovascular fitness, reduced burden of chronic disease and even improved mental health (Lavie et al., 2015; Oswald et al., 2020). Aside from the obvious health benefits, running is consistently one of the most popular sports due to being low cost, widely accessible, and its associated social engagement from running clubs and large running races (Clough et al., 1989; Janssen et al., 2020; Dejong et al., 2021). The implementation of COVID-19 restrictions meant that large races and group training in running clubs were often canceled, potentially having an impact on the motivation, participation, mental and physical health of runners during the pandemic.

Under the COVID-19 restrictions, running was one of the very few forms of exercise that could still be conducted due to its individual nature and the ease of maintaining social distancing restrictions. Two previous studies (Bazett-Jones et al., 2020; Dejong et al., 2021) examined the impact of COVID-19 restrictions on runners but identified conflicting results. Dejong et al. (2021) reported an increase in weekly running mileage (+1.4 km/week) and frequency (+0.3 session/week), whereas Bazett-Jones et al. (2020) found a decrease in the same variables, −5.4 km/week and −0.7 session/week respectively. The discrepancy between these two studies might be explained by the sampled populations. The severity and length of restrictions could be location-specific and could have affected the runners' training habits differently. Additionally, Bazett-Jones et al. (2020) focused exclusively on the impact in youth competitive runners, a population with unique challenges, running biomechanics and physiology (Krabak et al., 2021; McSweeney et al., 2021), thus further limiting the generalizability of these previous results. As such, the true impact of COVID-19 restrictions on adult runners' training habits is still unclear. Research into the impact of COVID-19 restrictions on physical activity habits will allow athletes, health professionals, and even public health officials, to better understand how population-wide infection control measures impact physical activity habits.

Both aforementioned studies (Bazett-Jones et al., 2020; Dejong et al., 2021) used a retrospective survey design, which allowed for quick analysis and identification of trends. However, a retrospective survey design increases the risk of recall bias (Hassan, 2006), mis-reporting of training variables and impacting on the accuracy of the results of the studies (Townshend et al., 2008; Dideriksen et al., 2016). A potential solution to this lies in the field of wearable technology. The increase in popularity and accessibility of wearable technology allows for a new way for researchers to collect real-world data on runners' training habits (Pobiruchin et al., 2017; Wiesner et al., 2018). Wearable devices, such as Global Positioning System (GPS) enabled smart watches, have recently been shown to be valid and reliable tool in tracking distance and heart rate among runners. These commercially available devices have shown a mean absolute percentage error of under 5% in tracking distance (Gilgen-Ammann et al., 2020), and a good to very good concurrent validity when tracking heart rate (Støve et al., 2019; Düking et al., 2020). Thus, wearable technology can be utilized as an alternative to runner surveys, collecting accurate, real-world data on running training variables for analysis.

Therefore, our study aimed to determine the impact of COVID-19 restrictions on runners' training habits using wearable technology. We aimed to determine the impact on running training variables, such as weekly mileage, frequency of training sessions, intensity of training sessions and duration of training. Based on Dejong et al. (2021) results in an adult population, we hypothesized that runners would have an increase in weekly mileage and frequency of training sessions. We also hypothesized that runners would have a reduced intensity of running training, due to reduced motivation from cancellation of races and running clubs (Dejong et al., 2021). Additionally, as most governments encourage outdoor activities during the COVID-19 pandemic, we also aimed to identify if runners had a change in the location of their running training. As exercise was one of the main reasons individuals were allowed outdoors during stay-at-home orders, we hypothesized that runners would have an increase in the number of training sessions performed outdoors during COVID-19 restrictions.

## Methods

### Participants

Data were extracted from the Wearable Technology Citizen Science Level-4 secure research database housed at the University of Calgary. This database contains activity records collected through a web portal (https://wetrac.ucalgary.ca) where participants for research projects can upload data from their GPS enabled smart watches. In the process of uploading their data, the runners gave permission for their data to be used in future research projects. The collection of data and study protocol were reviewed and approved by both the ethics board of the University of Calgary and Western Sydney University.

Retrospective training data, for the 24 months prior to participant sign up, and prospective weekly data, that had been collected since sign up, was then downloaded from the secure online database. The data were then screened for participants that met the inclusion criteria: (1) aged between 18 and 60; and (2) had data submitted from June 1, 2019 i.e., participants had at least 9 months of training data prior to the approximate start of COVID-19 restriction in March 2020. These criteria were selected to mitigate the unique demands of youth runners, such as lower rates and poorer recall of overuse injuries (Krabak et al., 2016, 2021), and the age-related decline in performance and training seen in older runners (Korhonen et al., 2003; Conoboy, 2006). The time period (i.e., 9 months prior to the start of COVID-19 restrictions) was set to ensure sufficient data for comparison before and after COVID-19 restrictions and mitigate the impact of seasonal running trends. Additionally, this time period ensured that runners had sufficient experience with using the GPS enabled smart watches, thus minimizing potential device errors such as incorrect wearing or data recording (Düking et al., 2020; Gilgen-Ammann et al., 2020).

The initial database search returned 112 potential participants and including devices from Garmin, FitBit, Suunto and Polar. These participants were contacted via email to confirm consent and demographic information. Following the screening, 40 participants were excluded as they did not reply to the email confirming consent, 2 participants were excluded as they did not have data available from before June 1, 2019, and a further 5 participants were excluded due to being over 60 years of age. Overall, a total sample size of *n* = 65 was used for the data analysis.

A power calculation was performed using an analysis of the data from *n* = 36 participants. Power calculation was performed in G^*^Power (Faul et al., 2007), using an alpha of 0.05, a power of 80% and an effect size of 0.4. We identified that our total sample size of *n* = 65 would be sufficient to power the study.

### Data Analyses and Processing

Data were downloaded from the secure database in the form of a MATLAB file for data processing. The brand and model of the wearable devices used by each participant was extracted. GPS location data from the GPS enabled smart watches was used to individualize the start date of COVID-19 restrictions based upon the local government restrictions (Table 1). We defined the start date of COVID-19 restrictions as the day that government mandated stay-at-home orders, closure of non-essential services, or other major restriction on the movement of people, were implemented.

**Table 1:** Implementation of COVID restrictions at participants' locations.

**Location**	**GPS coordinate**	**Date of COVID restriction implementation**
Auckland (New Zealand)	36° 52′ S, 174° 45′ E	March 25, 2020 (New Zealand Government, 2020)
Calgary (Canada)	51° 03′ N, 114° 05′ W	March 15, 2020 (Office of the Chief Medical Health Officer, 2020)
Denver (United States)	39° 44′ N, 104° 59′ W	March 26, 2020 (Governor of the State of Colorado, 2020)
Flagstaff (United States)	35° 11′ N, 111° 39′ W	March 30, 2020 (Ducey, 2020)
Grand rapids (United States)	42° 57′ N, 85° 40′ W	March 24, 2020 (The Office of Governor Gretchen Whitmer, 2020)
Hong Kong	22° 18′ N, 114° 10′ E	March 25, 2020 (The Government of the Hong Kong Special Administrative Region, 2020)
Kansas City (United States)	39° 05′ N, 94° 34′ W	March 24, 2020 (Lucus, 2020)
Los Angeles (United States)	34° 03′ N, 118° 14′ W	March 19, 2020 (Executive Department of The State of California, 2020)
Melbourne (Australia)	37° 50′ S, 144° 56′ E	March 16, 2020 (The Office of The Hon, 2020)
Milwaukee (United States)	43° 02′ N, 87° 02′ W	March 25, 2020 (City of Milwaukee, 2020)
Scranton (United States)	41° 24′ N, 75° 39′ W	March 20, 2020 (Commonwealth of Pennsylvania, 2020)
Dallas (United States)	32° 46′ N, 96° 48′ W	March 19, 2020 (Governor of the State of Texas, 2020)
Toronto (Canada)	43° 39′ N, 79° 20′ W	March 17, 2020 (Office of the Premier, 2020)

Descriptive data on age, gender, location, and average pace in minutes per kilometer were collected. Each participant's average pace, in minutes per kilometer, was compared to age matched norms using an online calculator (Grubb, 2015), that compares individual performance to historical race records. The age-matched norms returned a percentile value, which was interpreted as; recreational runners <60th percentile, and competitive runners ≥ 60th percentile. The use of age-matched norms is a common method of identifying competitive performance of runners, as it is regularly used by the US Masters Track and Field Association during competitions (USTAF Masters Organisation, 2018), as well as in previous studies relating to identifying running performance (Clermont et al., 2019; Liu et al., 2020).

We then analyzed the impact on the COVID-19 restrictions on the selected running training variables: average weekly running distance (km/week), average frequency of training sessions per week (number per week), the average distance of each individual run (km), the average duration of each running training session (minutes) and the average intensity of training sessions (percentage of maximal heart rate). Percentage of maximal heart rate was calculated as:


$$\begin{array}{l} Percentage\;of\;Maximal\;Heart\;Rate\;=\frac{Average\;Heart\;Rate}{220\;-\;Age}\times \;100 \end{array}$$

The formula used to calculate maximal heart rate percentage is from the American College of Sport Medicine guidelines on exercise prescription, and is a common method of measuring the intensity of training sessions (American College of Sports Medicine, 2018). To address our second research aim, we counted the average number of outdoor runs per week. A run was considered outdoors if the user recorded the run as “Running,” “Track running,” “Street running,” or “Trail running.”

High intensity interval training (HIIT) has gained popularity with runners over recent years due to its proposed shorter training volumes with equivalent fitness gains when compared to steady state aerobic training (Foster et al., 2015). Runners that had recorded ≥ 3 runs, of distances <800 m, and within the space of 1 h, were considered to have participated in a HIIT session (Gibala et al., 2012). As such, to prevent an over-estimation in weekly frequency and distances, the training variables of distance and time for each run as part of a HIIT session were added together to form a single training session.

Following processing, the data was analyzed using SPSS (version 27.0, IBM Corp., Armonk, NY, USA). Shapiro-wilk tests were used to assess for data normality, with dependent samples *t*-tests used to assess for significance. Effect size was calculated using Cohen's *d*, and interpreted as 0.2 = small effect, 0.5 = moderate effect and 0.8 = large effect. An effect size smaller than 0.2 was interpreted as trivial (Cohen, 1988).

## Results

**Figure 1:**
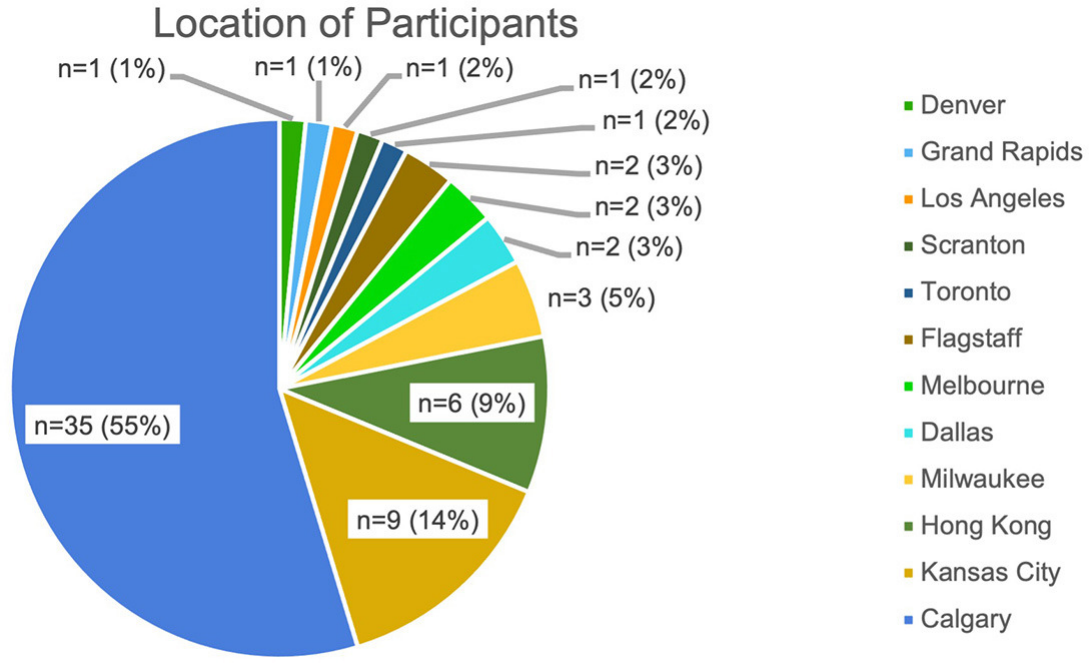
The locations of participants included in the study.

The average age for participants was 38.1 years (±10.5 years), with *n* = 40 (63.1%) being males. Participants were recruited from 5 different countries, in 11 different cities (Figure 1). A majority of participants (55%) were from Calgary in Canada. The average pace per kilometer was 6 mins and 6 s, which was equivalent to the 38th percentile for age-matched norms. Additionally, no participants were above the 60th percentile, indicating our study exclusively examined recreational runners.

A total of 101 devices were used by the participants, with 54% of the participants using one device throughout the time period. There were 38, 6 and 2% of the participants using two, three and four devices respectively. All the devices were manufactured by Garmin (Olathe, KS, USA). The distribution of device models are presented in Table 2.

**Table 2:** Distribution of wearable devices used by participants.

**Series**	**Model**	***n***
**Forerunner®**		**50**
	235	9
	735 XT	8
	935	6
	220	5
	230	4
	920 XT	4
	245	2
	245M	2
	645	2
	945	2
	310T	1
	35	1
	630	1
	645M	1
	745	1
	910 XT	1
**fēnix®**		**21**
	5s	7
	3 HR	4
	5x	4
	6x Pro Solar	2
	3	1
	6	1
	6 Pro	1
	6s	1
**v**í**voactive®**		**20**
	3	7
	1 (Original)	4
	3 Music	4
	HR	4
	4 Wifi	1
**Others**		**10**
	Venu®	4
	Instinct®	2
	MARQ® Captain	1
	tactix® Delta	1
	vívosport®	1
	unidentified	1

Overall, we found that runners had statistically significant but trivial increases in their average weekly mileage [mean difference = 3 km, 95% confidence interval (CI) 0.02–5.9 km, *p* = 0.05] and number of training sessions per week (mean difference = 0.3 session, 95% CI 0.02–0.65 session, *p* = 0.33). Also, we found a small-to-moderate increase in the number of outdoor training sessions runners participated in each week (mean difference = 0.47 session, 95% CI 0.16–0.71 session, *p* = 0.003). We found that COVID-19 restrictions had no significant impact on the intensity of the running session (*p* = 0.677). The summary of the results can be seen in Table 3.

**Table 3:** The impact of COVID-19 restrictions on running training variables before and the implementation of restrictions.

**Training variables**	**Pre-COVID-19**	**During COVID-19**	**Cohen's *d***	***p***
Weekly mileage (km)	25.0 ± 23.7	28.0 ± 25.3	0.12	0.05^*^
Distance per run (km)	7.3 ± 2.7	7.4 ± 2.7	0.03	0.64
Duration per run (min)	45.1 ± 16.9	46.6 ± 14.8	0.12	0.33
Number of trainings per week	3.0 ± 2.4	3.4 ± 2.5	0.14	0.03^*^
Intensity (% of maximal heart rate)	79.2 ± 6.9	78.8 ± 7.1	0.05	0.72
Number of outdoor runs per week	2.6 ± 2.2	3.1 ± 2.3	0.21	<0.01^*^
Percentage of outdoor run (%)	86.1 ± 22.2	93.0 ± 19.9	0.33	0.01^*^

* *Significance at p < 0.05*.

## Discussion

The purpose of our study was to determine the impact of COVID-19 restrictions on runners' training habits, including frequency, intensity and duration of training, weekly mileage and running locations. We found that runners adjusted their training habits during the COVID-19 pandemic, which supports our primary hypotheses of increased weekly mileage and number of runs per week. However, no differences were found in the intensity and duration of the training sessions. Results also demonstrated an increase in the percentage and number of outdoor runs, which also aligned with our hypothesis.

Overall, participants of our study increased their weekly mileage by an average of 3 km. This statistically significant increase, although trivial, has demonstrated participants' adherence to running even under COVID-19 restrictions. A similar increase of 1.4 km was reported in Dejong et al. (2021) large-scale survey study, the current study observed a larger increase, possibly attributed to the difference in the reporting time frame and methods employed between the two studies. Specifically, DeJong et al.'s study compared running habits before the pandemic and at peak social isolation restriction (May to June 2020), while we averaged the weekly mileage across 9 months before and after restrictions (March 2020) were introduced. COVID-19 has disrupted not only physical activity, but mental health, family life and daily lifestyle (Petersen et al., 2021) and a longer time frame could have allowed runners to overcome the stress, adapt to the changes and restore their routines, including running trainings. This supposition is supported by a recent study that reported an initial decline in physical activity engagement within the first month of COVID-19 lockdown, but engagement was restored and even surpassed the level before COVID-19 restrictions after 3 months (Mata et al., 2021). Future studies should consider longitudinal monitoring of a homogenous cohort to further examine effect on both running behaviors and injury risk of COVID-19 restrictions and the lifting of such restrictions.

In addition to the increased weekly mileage, an increased number of runs per week was also observed among participants involved in the current study. The training duration remained unchanged, suggesting that runners were running more frequently rather than completing longer runs. Longer runs has been associated with fatigues and potentially injurious biomechanics (Futrell et al., 2021). Considering the trivial increase in weekly mileage, frequency and duration of runs, the relative risk of running-related injury is likely unchanged. Moreover, intensity of runs were also unchanged, most likely due to most marathon races in 2020 being canceled or postponed, including major races such as the Boston Marathon (Minsberg and Futterman, 2020) and the Berlin Marathon (Bussiness Standard, 2020). Bazett-Jones et al. reported that changes to training intensity were especially prominent within competitive youth long-distance runners (Bazett-Jones et al., 2020). However, in the current study, participants were adults who ran an average of 25 km per week before the pandemic. This result is further supported by the fact that runners in the current study maintained their percentage of maximal heart rate at 72–86%, which is considered moderate-to-vigorous intensity physical activity, for around 135 mins per week. This amount of moderate-to-vigorous intensity physical activity met the recommendations for substantial health benefits according to the guidelines on exercise prescription published by the American College of Sports Medicine (2018) and further supports the shift in motivation from performance to maintaining physical activity level and health benefits (Dejong et al., 2021; Petersen et al., 2021).

A significant increase in the number of outdoor runs and the percentage of outdoor runs was also observed in the current study. This finding aligns with Dejong et al. (2021) and can be explained by two main reasons—the closure of fitness centers and indoor running tracks and governments encouraging outdoor physical activities. For example, the majority (55%) of the runners in our study reside in Calgary, Canada and the provincial government declared a state of public health emergency on March 15, 2020 (Office of the Chief Medical Health Officer, 2020) and implemented a 3-month closure of sport programs, fitness centers and recreation facilities, which likely forced runners to run outdoors. Various states in the United States and Hong Kong have also implemented similar preventive measures, enforcing phased closure of fitness centers since March 2020 (Fisher, 2021). Furthermore, the government has also encouraged outdoor physical activities (Government of Alberta, 2020) for the possibility of socially distancing and limiting indoor airborne transmission of COVID-19 (Bazant and Bush, 2021). Additionally, spending more daytime outdoors has also been found to extenuate the negative effect of COVID-19 restrictions and prevent sleep disruption (Korman et al., 2021).

Our study is the first to our knowledge to implement the use of GPS enabled wearable devices to evaluate training habits in response to COVID-19 restrictions among runners. Recall bias on running pace and training frequency (Townshend et al., 2008; Nielsen et al., 2012) and self-reported distance (Dideriksen et al., 2016) associated with the use of surveys and diaries were overcome thereby allowing more reliable, objective and quantitative evaluation of changes in training habits. The current study further supports the findings of other qualitative and large-scale survey studies conducted on the impact of COVID-19 restrictions. Furthermore, our study has established the framework for using wearable devices to evaluate individual or group level changes across longer time periods. Regardless, limitations should be acknowledged.

A limitation of the current study was the lack of characteristic information such as years of running experience, self-reported level and injury conditions among the participants. The online portal only registers training data that were recorded by the wearable device, and we have only obtained the age and gender of participants. Further sub-group analysis was therefore unavailable based on the existing dataset. Another limitation is that the devices used for recording heart rate are all wrist-worn Garmin devices, which was found to have a higher mean absolute percentage error than electrocardiogram measured by a chest-band (Evenson and Spade, 2020). Lastly, the uneven distribution of physical location among the participants could limit the generalizability of our results. Majority of runners from North America (United States and Canada). Future studies involving similar methodologies may bring new insights into how specific groups of runners altered their running habits as a response to COVID-19 restrictions.

In conclusion, COVID-19 restrictions have brought changes to runners' training habits. Compared to pre-COVID-19, runners have increased their weekly mileage, frequency, and outdoor training sessions during the pandemic. Coaches and sport medicine clinicians should consider the impact that COVID-19 restrictions may have on training-related risk factors of overuse injuries, and advise runners on plans to ensure safe changes to their training habits.

## Data Availability Statement

The raw data supporting the conclusions of this article will be made available by the authors, without undue reservation.

## Ethics Statement

The studies involving human participants were reviewed and approved by Ethics Board of the University of Calgary and Western Sydney University. The participants provided their written informed consent to participate in this study.

## Author Contributions

RC, RF, and GC contributed to the conception of the study. ZC, RP, and RC contributed to the experimental setup, conducted statistical analyses, and drafted the manuscript. ZC and RP conducted the participant recruitment, data acquisition, and processing. ZC, RP, GC, RF, and RC revised the article for important intellectual content. All authors approved the final version.

## Funding

This study was partially funded by the NSERC CREATE Wearable Technology Research and Collaboration (We-TRAC) Training Program (Project No. CREATE/511166-2018), a City of Calgary Council Innovation Fund (Project No. 1053228) and a grant from the Calgary Foundation (Project No. 20200518).

## Conflict of Interest

The authors declare that the research was conducted in the absence of any commercial or financial relationships that could be construed as a potential conflict of interest.

## Publisher's Note

All claims expressed in this article are solely those of the authors and do not necessarily represent those of their affiliated organizations, or those of the publisher, the editors and the reviewers. Any product that may be evaluated in this article, or claim that may be made by its manufacturer, is not guaranteed or endorsed by the publisher.
